# Synthesis of Alginate Nanoparticles Using Hydrolyzed and Enzyme-Digested Alginate Using the Ionic Gelation and Water-in-Oil Emulsion Method

**DOI:** 10.3390/polym15051319

**Published:** 2023-03-06

**Authors:** Nicolas Van Bavel, Anna-Marie Lewrenz, Travis Issler, Liping Pang, Max Anikovskiy, Elmar J. Prenner

**Affiliations:** 1 Department of Biological Sciences, University of Calgary, Calgary, AB T2N 1N4, Canada; 2 Institute of Environmental Science and Research, P.O. Box 29181, Christchurch 8540, New Zealand; 3 Department of Chemistry, University of Calgary, Calgary, AB T2N 1N4, Canada

**Keywords:** nanoparticle synthesis, biopolymers, alginate, ionic gelation, water-in-oil emulsification, scanning electron microscopy

## Abstract

Alginate nanoparticles (AlgNPs) are attracting increasing interest for a range of applications because of their good biocompatibility and their ability to be functionalized. Alginate is an easily accessible biopolymer which is readily gelled by the addition of cations such as calcium, facilitating a cost-effective and efficient production of nanoparticles. In this study, AlgNPs based on acid hydrolyzed and enzyme-digested alginate were synthesized by using ionic gelation and water-in-oil emulsification, with the goal to optimize key parameters to produce small uniform (<200 nm) AlgNPs. By the ionic gelation method, such AlgNPs were obtained when sample concentrations were 0.095 mg/mL for alginate and CaCl_2_ in the range of 0.03–0.10 mg/mL. Alginate and CaCl_2_ concentrations > 0.10 mg/mL resulted in sizes > 200 nm with relatively high dispersity. Sonication in lieu of magnetic stirring proved to further reduce size and increase homogeneity of the nanoparticles. In the water-in-oil emulsification method, nanoparticle growth was confined to inverse micelles in an oil phase, resulting in lower dispersity. Both the ionic gelation and water-in-oil emulsification methods were suitable for producing small uniform AlgNPs that can be further functionalized as required for various applications.

## 1. Introduction

Nanoparticles (NPs) formed from natural biopolymers are of great interest for biomedical, pharmaceutical, food, industrial, agricultural, and environmental applications. Natural biopolymers are biocompatible, non-toxic, biodegradable, readily available, and their physiochemical properties can be easily modified [1,2,3,4]. One abundant natural biopolymer is alginate. Alginate NPs have shown great promise in their applications in drug delivery, environmental protection (e.g., water treatment, environmental-friendly fertilizers, herbicides, and pesticides), and antimicrobial food additives and packaging [5,6,7,8,9,10,11].

**Figure 1:**
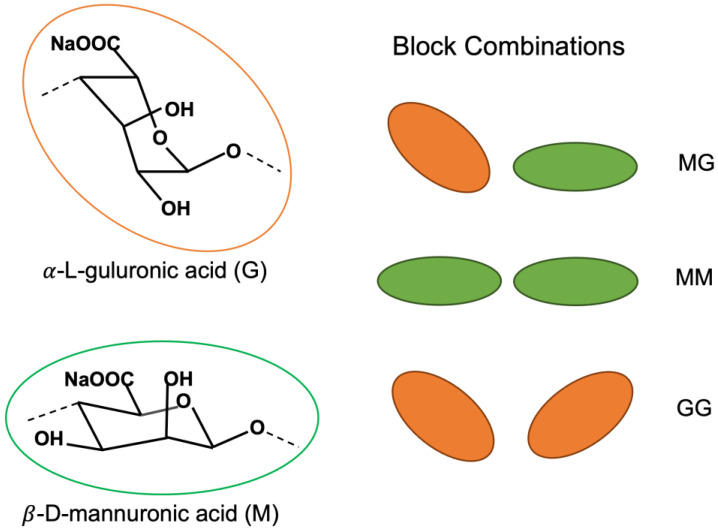
Schematic of uronic acids their arrangements in homo- and heteropolymeric blocks.

Alginate is a hydrophilic anionic polysaccharide derived from brown algae and bacteria. It is a copolymer of 1–4 linked *β*-D-mannuronic acid (M) and *α*-L-guluronic acid (G), forming homo-polymeruronic acid blocks (MM-block and GG-block) and hetero-polymeruronic acid blocks (MG-block) (Figure 1) [12,13]. In order to produce small (<200 nm) alginate NPs (AlgNPs) and alginate nanocomposites with low polydispersity, alginate is sometimes broken down into short chains of uronic acid so that the homo-polymeric fractions may be extracted for the use of AlgNPs synthesis [14,15]. Haug et al. performed the first partial hydrolysis of alginate in 1966, and his method has been adapted over the years, allowing researchers to isolate MG, MM, and GG blocks in individual fractions based on hydrolysis time and the solubility of each acid at a given pH [14,16,17,18]. In addition to acid hydrolysis [13,14,19,20], alginate can be broken down by alkali hydrolysis [19] or enzymatic digestion using the enzyme alginate lyase [18,21].

The size of NPs influences NP interactions with biological systems and environmental media and affects the effectiveness of antimicrobial food packaging [22,23,24,25,26]. It was reported that the size range of 10–200 nm NPs is most relevant to physical and biochemical targeting through both intravascular and site-specific deliveries [27]. Although many studies have been published on AlgNPs synthesis, as summarized in the reviews [28,29], the majority of these studies report synthesis of AlgNPs with sizes > 200 nm. Of the limited number of studies that have reported synthesis of AlgNPs with sizes < 200 nm, most articles do not provide detailed experimental procedures, which makes reproducing the experimental results difficult or impossible.

Two common methods used for AlgNPs synthesis are ionic gelation and water-in-oil emulsification [28,29,30,31,32]. The former involves crosslinking anionic polymer units with a cation, generally Ca^2+^. The latter method also makes use of this gelation process, but the aqueous phase required for NP growth is confined within inverse micelles in an oil phase (water-in-oil). This method allows for better control over NP size and uniformity, as these are limited by the size of the inverse micelles.

The aim of this study was to produce uniform AlgNPs with size < 200 nm by optimizing the experimental parameters for both the ionic gelation and water-in-oil emulsification methods. Alginate breakdown with acid hydrolysis was compared to the enzyme alginate lyase in terms of AlgNPs size and size distribution. Finally, the efficacies of AlgNPs synthesis and their physical characteristics were compared between the two synthesis methods.

## 2. Materials and Methods

### 2.1. Materials

Sodium alginate was purchased from Acros Organics (Geel, Belgium). Calcium chloride dihydrate (CaCl_2_•2H_2_O) was purchased from Fisher Scientific (Nepean, ON, Canada). Alginate lyase (≥10,000 units/g), bis-(2-ethylhexyl) sulfosuccinate sodium salt (AOT), toluene, ethanol (95%), acetone, and hydrochloric acid were purchased from Sigma-Aldrich (Markham, ON, Canada).

### 2.2. Breakdown of Alginate

#### 2.2.1. Acid Hydrolysis

**Figure 2:**
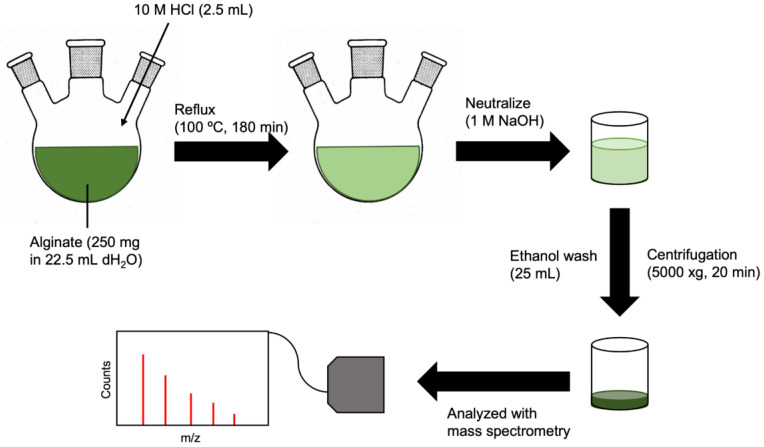
Schematic of alginate breakdown by acid hydrolysis.

The breakdown of alginate by acid hydrolysis was adapted from Daemi and Barikani [14]. Briefly, sodium alginate (250 mg) was dissolved in dH_2_O (22.5 mL) in a 100 mL-round bottom flask (Figure 2). The solution was heated to 50 °C under constant stirring (800 rpm) to ensure complete dissolution of alginate. This was followed by an injection of 10 M HCl (2.5 mL) to a final concentration of 1 M. The solution was refluxed at 100 °C for 180 min, neutralized with 1 M NaOH, and washed twice with ethanol. The product was isolated with centrifugation (5000× *g*, 20 min).

#### 2.2.2. Alginate Lyase

The digestion of alginate-by-alginate lyase was adapted from Ochi et al. [18]. First, the alginate lyase solution was prepared to an activity of 10 units/mL by suspending 8.3 mg of alginate lyase (≥10,000 units/g) in 8.3 mL of phosphate buffer (1 mM K_2_PO_3_, 10 mM NaCl, 0.02% NaN_3_, pH 6.3). This buffer was chosen to optimize the enzyme activity. A 25 mL NaCl solution (0.1 M, pH 8.0) was heated to 50 °C, followed by a portion-wise addition of 250 mg sodium alginate over the course of 2 h, to a final concentration of 1% *w*/*v*. The heat was then reduced to 37 °C and the alginate lyase buffer solution was added (8.3 mL). The suspension was incubated at 37 °C for 24 h under magnetic stirring (300 rpm). The alginate product was washed twice with ethanol and isolated by centrifugation (4696× *g*, 20 min, 4 °C), then dried in a vacuum oven overnight (40 °C).

Prior to AlgNPs synthesis, the alginate precursor solution was filtered through a VivaSpin 2, 5000 MWCO spin column (VivaProducts, Littleton, MA, USA) at 4000× *g* for 60 min. The flow-through was filtered with a VivaSpin 2, 2000 MWCO spin column (VivaProducts, Littleton, MA, USA) at 4000× *g* for 40 min to remove undigested polymer. The concentration of the resulting filtrate was determined by the drying and weighing of a 50 μL aliquot.

#### 2.2.3. Mass Spectrometry

Mass spectrometry was used to analyze the breakdown products of alginate by acid hydrolysis and alginate lyase, determining the degree of hydrolysis. Positive electrospray ionization mass spectrometry (+ESI-MS) (Agilent, Santa Clara, CA, USA) was used for samples from acidic hydrolysis and negative ESI-MS for enzymatic digestion.

### 2.3. Alginate Nanoparticle Synthesis and Characterization

#### 2.3.1. Ionic Gelation

**Figure 3:**
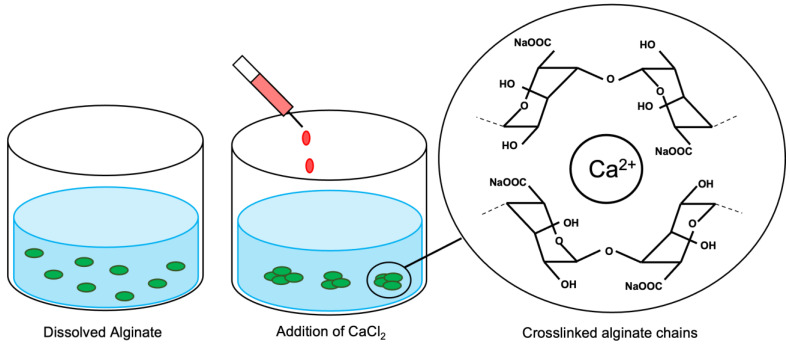
Schematic of AlgNPs synthesis using the ionic gelation method.

Ionic gelation-based synthesis utilizes electrostatic forces to crosslink polymer units of interest, leading to the spontaneous formation of NPs (Figure 3). Here, negatively charged alginate was used as the polymer, while calcium ions were added as the crosslinker. The formation of AlgNPs in a controlled manner depends on the concentration of both alginate and calcium, as well as the homogenization speed and time of the synthesis process. The effects of altering alginate and calcium concentrations and volumes were tested, as well as the use of either magnetic stirring, bath sonication, or a homogenizer for mixing of the sample.

The protocol of ionic gelation was adapted from Daemi and Barikani [14] and Saraei et al. [31], whereby the acid hydrolyzed or enzyme-digested alginate was first dissolved in dH_2_O (9.5 mL). A stir plate at 1300 rpm, a bath sonicator (FS110H, Fisher Scientific, Nepean, ON, Canada), and a handheld homogenizer motor at 7000 rpm (Fisherbrand^TM^ 150, Fisher Scientific, Nepean, ON, Canada) were compared in terms of their homogenization effect to produce monodisperse AlgNPs in the selected size range. The CaCl_2_ solution (0.5 mL) was added dropwise (~1 mL/min), and the mixture was left for 45–60 min. After the allotted time, the particles were washed, collected by centrifugation (20,000× *g*, 40 min), and resuspended in ddH_2_O. This synthesis was performed for various concentrations of alginate (0.095–1.2 mg/mL) and CaCl_2_ (0.03–2.7 mg/mL) (Table 1) to find the most suitable reagent ratio.

#### 2.3.2. Water-in-Oil Emulsification

**Figure 4:**
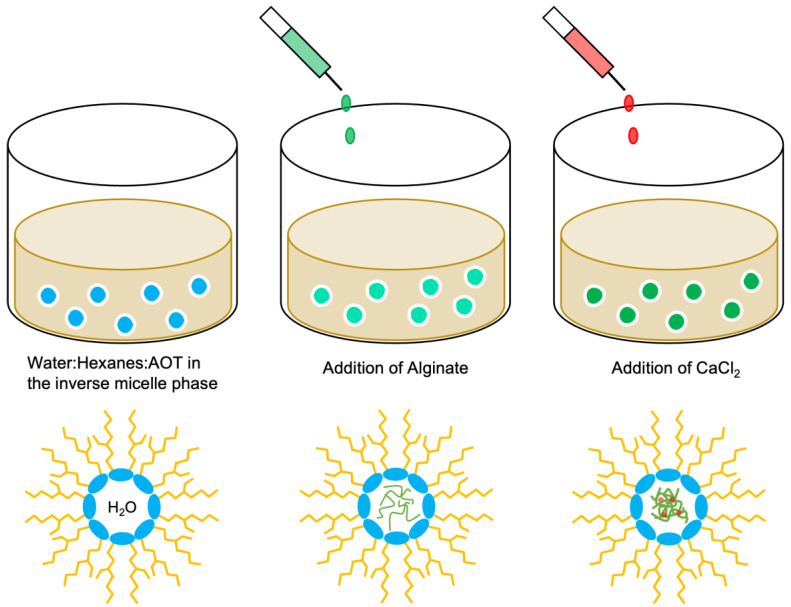
Schematic of AlgNPs synthesis using the water-in-oil emulsification method.

In the process of water-in-oil emulsification, the addition of an aqueous solution to an organic solution produces water droplets in the organic phase that can be stabilized by a surfactant (Figure 4). The water-soluble alginate accumulates in these droplets. Consequently, the size of the nanoparticles is defined by the size of the droplets. Therefore, the formation of <200 nm AlgNPs will require an emulsion of water droplets of similar size. Subsequently, CaCl_2_ was added to crosslink the alginate and stabilize the particles. The surfactant and organic solvent can be separated from the aqueous phase, yielding uniform AlgNPs.

The protocol for the water-in-oil emulsification method was adapted from You and Peng (2005) [32]. In order to form the inverted micelle phase, a 20:40:40 water:hexanes:AOT weight percent ratio was used. Hexanes (1.215 mL), dH_2_O (0.40 mL), and (AOT) (0.8 g) were added in the corresponding order and the solution was mixed with the Fisherbrand^TM^ mini tube rotator (15 rpm, 20 min). Enzyme-digested alginate solution (25 mg/mL, 50 μL) were then added to the emulsion and mixed on a Fisherbrand^TM^ mini tube rotator (10 min, 15 rpm). Next, CaCl_2_ (60 mg/mL, 50 μL) was added and the emulsion was mixed on the tube rotator (10 min, 15 rpm). In order to wash and collect the AlgNPs, acetone (3 mL) was added dropwise followed by centrifugation (4696× *g*, 20 min). The pellet was resuspended in ddH_2_O (1 mL), and ethyl acetate was added at a 2:1 volume ratio and vortexed (30 s). The upper organic solvent layer was removed, while the lower aqueous layer containing AlgNPs was washed again with ethyl acetate before filtration through a 0.2 μm pore size polyethersulfone syringe filter (J.T.Baker^®^, Avantor, Radnor Township, PA, USA).

#### 2.3.3. Alginate Nanoparticle Characterization

AlgNPs size and polydispersity index (PDI) were determined by dynamic light scattering using a Zetasizer Nano ZS (Malvern, UK). All measurements were performed in triplicate at 25 °C (10 runs per measurement, 10 s/run) in disposable polystyrene zeta cuvettes.

The imaging of AlgNPs was done with a scanning electron microscope (Zeiss Sigma VP, Field Emission, Zeiss, Heidenheim, Germany) using an InLens. Samples (50 µL) were deposited on silicon wafers and dried under nitrogen prior to imaging.

## 3. Results and Discussion

### 3.1. Alginate Hydrolysis

The presented protocols yielded relatively uniform AlgNPs with sizes < 200 nm that are promising scaffolds in a biologically relevant range for further functionalization and were development as described recently [33].

**Figure 5:**
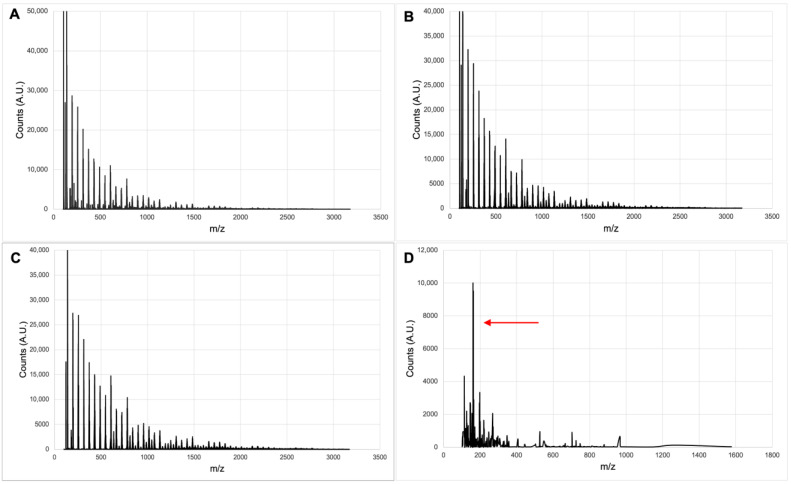
Electrospray ionization mass spectrometry of alginate samples digested with (**A**) 1 M HCl for 3 h, (**B**) 2 M HCl for 3 h, (**C**) 3 M HCl for 3 h, and (**D**) Alginate lyase for 24 h. The red arrow indicates signal at 158 *m*/*z*.

Figure 5 shows mass spectrometry results of alginate hydrolysis using HCL acid and alginate lyase. Acidic hydrolysis yielded a similar mass-to-charge ratio (*m/z*) distribution for 1, 2, and 3 M HCl after 3 h (Figure 5A–C). The steps between major peaks differed by 58 *m*/*z*, and applying a charge of +3 gives a weight difference of 174 Da, which is the approximate weight of a single monomer hydrolyzed from the polymer chain [21]. From this, the size range of the fragments was determined to be between 590 Da to 5500 Da (3 monomers/chain—28 monomers/chain). Contrarily, enzymatic digestion of alginate by alginate lyase yielded a high amount of product at 158 Da, the weight of monomer units after the *β*-elimination reaction [34], with relatively few larger fragments compared to acidic hydrolysis (Figure 5D).

Table 1 shows that full acid hydrolysis of alginate for synthesis resulted in AlgNPs (samples IG1, IG2, IG3) of a similar size (206–242 nm) and comparable uniformity (PDI = 0.155–0.178). This is consistent with the findings of Daemi and Barikani [14], who reported that partial acid hydrolysis and isolation of MM block supported greater uniformity of AlgNPs. Furthermore, enzymatic digestion with alginate lyase provided a more suitable precursor for the synthesis of smaller AlgNPs (108–200 nm) than those from the acid hydrolysis (206–242 nm) (Table 1, IG1 vs. IG5 and IG10; IG2 vs. IG7). This can be attributed to a high yield of monomer units (158 Da) from the enzymatic digestion processes, as determined by mass spectrometry (Figure 3D, red arrow). Therefore, enzyme-digested alginate is recommended for future use as a polymer precursor in AlgNPs synthesis.

### 3.2. Alginate Nanoparticle Synthesis

#### 3.2.1. Ionic Gelation

The sizes of AlgNPs produced by ionic gelation ranged between 108–242 nm at alginate concentrations ≤ 0.57 mg/mL and CaCl_2_ ≤ 0.1 mg/mL (Table 1, IG1–IG7, IG10), with PDI ≤ 0.178 except for IG4 (PDI = 0.617) and IG5 (PDI = 0.695). This size range was close to our proposed target size of ≤200 nm. When the alginate concentration was >0.57 mg/mL or/and CaCl_2_ > 0.1 mg/mL, and sizes of AlgNPs were >300 nm except for IG6 (*n* = 232 nm, PDI = 0.072).

The optimal final concentrations of alginate and CaCl_2_ to produce uniform AlgNPs < 200 nm were 0.095 mg/mL alginate and 0.1 mg/mL CaCl_2_ (IG4, IG5, IG10), except for IG1 (*n* = 227.6 nm). At 0.1 mg/mL CaCl_2_, higher alginate concentrations (0.285–2.7 mg/mL) increased particle sizes to >200 nm (IG2, IG3, IG7, IG12, IG14), although sizes of IG3 (*n* = 206 nm) and IG7 (200 nm) were comparable to the desired size.

The average chain length of the alginate precursor is an important factor in producing uniform AlgNPs with sizes < 200 nm. The smallest size of AlgNPs (108 nm; Table 1, IG10) was obtained with 0.095 mg/mL of enzyme-digested alginate and 0.1 mg/mL CaCl_2_ homogenized in a bath sonicator (Fisher Scienticfic, FS110H, Nepean, ON, Canada) for 60 min.

The use of acid hydrolyzed alginate precursor at alginate concentrations (0.095–0.570 mg/mL) and 0.1 mg/mL CaCl_2_ resulted in consistent particle sizes of >200 nm (Table 1 IG1, IG2, IG3), although IG3 (206 nm) was close to 200 nm. In contrast, when enzyme-digested alginate was used as the polymer precursor, AlgNPs were <200 nm (IG4, IG5, IG10) at alginate 0.095 mg/mL and CaCl_2_ ≤ 0.1 mg/mL. Likewise, at the same concentrations of alginate 0.285 mg/mL and CaCl_2_ 0.1 mg/mL, smaller AlgNPs were produced with enzyme-digested alginate (200 nm IG7) than AlgNPs with acid hydrolyzed alginate (242 nm IG2).

The homogenization method also influenced AlgNPs size and PDI. Increasing alginate concentration at higher CaCl_2_ concentrations (>0.28 mg/mL) increased particle size under magnetic stirring (IG6, IG9), but did not have an impact on size under bath sonication (IG11, IG13). Therefore, utilizing bath sonication for homogenization during synthesis may allow obtaining smaller uniform AlgNPs. At the same concentrations of enzyme-digested alginate (0.095 mg/mL) and CaCl_2_ (0.1 mg/mL), AlgNPs homogenized using a bath sonicator were smaller and more uniform (IG10 108 nm, PDI 0.165) than those using the magnetic stirring (IG5 118.3 nm, PDI = 0.695). The use of a Fisherbrand^TM^ 150 handheld homogenizer motor resulted in distinct populations of AlgNPs arising in the sample (Table 1, IG14). The median size was 722 nm and made up 41.2% of the population. A smaller population was measured to have a size of 96 nm at 21.1% of the population. In order to isolate this smaller population, ultracentrifugation may be used. Alternatively, the sample can be passed through a membrane filter with a pore size of 0.1 μm. However, as the use of a homogenizer allows the production of AlgNPs < 100 nm, the concentrations of the precursors may need to be decreased in order to increase the homogeneity of this sample.

**Figure 6:**
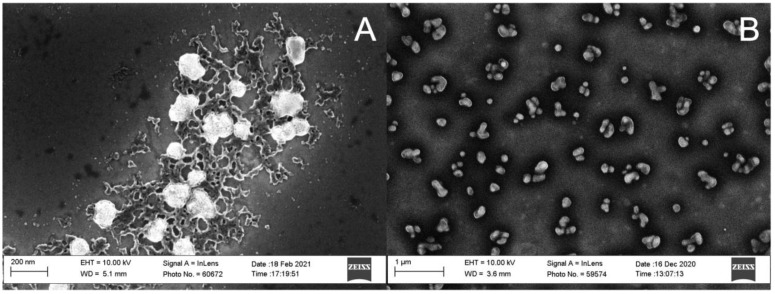
Scanning electron microscope images of alginate nanoparticles synthesized using enzyme-digested alginate with the ionic gelation method. (**A**) 0.10 mg/mL alginate and 0.10 mg/mL Ca^2+^ homogenized with a bath sonicator (IG10). (**B**) 0.10 mg/mL alginate and 0.10 mg/mL Ca^2+^ homogenized with a magnetic stir plate at 1300 rpm (IG5).

Scanning electron microscope (SEM) images confirmed the <200 nm size of AlgNPs synthesized with enzyme-digested alginate 0.095 mg/mL and CaCl_2_ 0.10 mg/mL, homogenized in a bath sonicator (Figure 6A, Table 1 IG10). Additionally, a gel-like structure was observed amongst the AlgNPs (Figure 6A). These are likely remnants of hydrogels that commonly form during the gelation processes [35]. Given that alginate and calcium ions are readily available in solution, continuous crosslinking into micro and macro structures is expected. While such particles have been used in the biomedical industry [35], their presence does not support the aim to synthesize homogenous AlgNPs.

SEM images revealed the high disparity of AlgNPs synthesized with enzyme-digested alginate 0.095 mg/mL and CaCl_2_ 0.10 mg/mL, homogenized with magnetic stirring (Figure 6B). Uncontrolled nanoparticle growth may have occurred, leading to a disparity in NP sizes as reflected by high polydispersity (0.695, IG5). This may be overcome with changes to ionic strength and pH of the solution, as demonstrated for NP synthesis based on the positively charged polymer chitosan [36,37]. Moreover, the homogenization method is an important factor to prevent uncontrolled growth of nanoparticles. The use of a bath sonicator in lieu of magnetic stirring further increased uniformity and decreased particle size at equal alginate and CaCl_2_ concentrations (Figure 4A,B).

#### 3.2.2. Water-in-Oil Emulsification

The above-mentioned problem of hydrogel formation may be avoided by using the water-in-oil emulsification method. This is because AlgNPs size and uniformity can be controlled more directly through the size of the inverse micelles formed in the emulsion.

**Figure 7:**
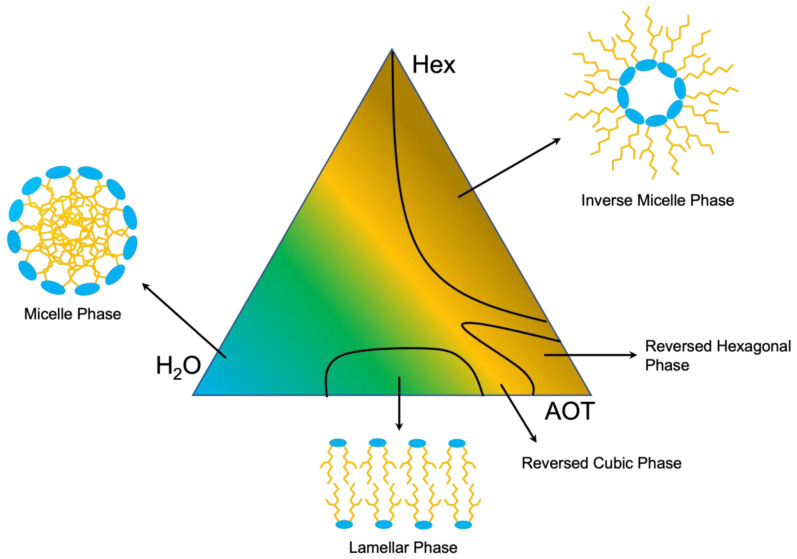
Phase diagram of water:hexanes:AOT used in the water-in-oil emulsification method.

The phase landscape formed by ternary mixture of water:organic phase:surfactant was first explored by altering these ratios according to Mesa et al. [38], and a variety of phase structures were reported. In order to obtain a well-defined and reproducible separation, the formation of an inverted micelle phase was targeted (Figure 7). A weight ratio of 40:40:20 (water:hexanes:AOT) proved to produce a stable and reproducible outcome, and this ratio was within the range of the inverted micelle phase reported previously [38]. While a significant number of ratios were tested, only the ideal mixture was included in Table 2.

**Figure 8:**
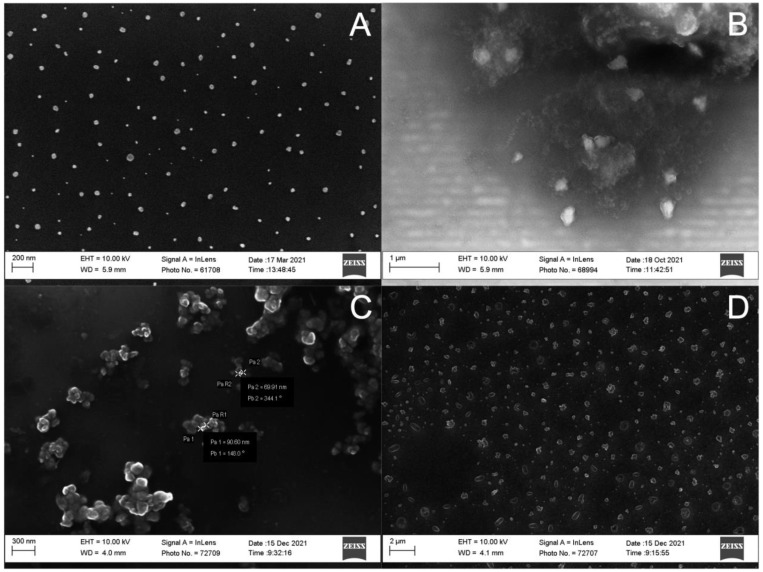
Scanning electron microscope images of alginate nanoparticles synthesized with the water-in-oil emulsification method. (**A**): 0.5 mg/mL alginate and 0.6 mg/mL Ca^2+^ (E1). (**B**) 2.5 mg/mL alginate and 0.6 mg/mL Ca^2+^ (E3). (**C**) 2.5 mg/mL alginate and 0.6 mg/mL Ca^2+^ with ethyl acetate wash (E7). (**D**) 5.0 mg/mL alginate and 0.6 mg/mL Ca^2+^ (E8).

After the addition of enzyme-digested alginate (5 mg/mL, 50 μL), and CaCl_2_ (60 mg/mL, 50 μL), AlgNPs with an average size as low as 138 nm and a PDI of 0.210 were collected (Table 2 E1). Size analysis by SEM revealed a size of 60 nm (Figure 8A). A smaller size for SEM was expected, as this measurement does not include the hydration shell of the particles in contrast to the hydrodynamic radius reported by the Zetasizer. Repeated syntheses produced slightly larger particles and higher amounts of residual surfactant AOT was visible under the SEM. In order to increase reproducibility and purity of the AlgNPs, various purification reagents were tested to wash AlgNPs after the emulsion step.

Acetone and isopropanol washes were conducted to isolate the AlgNPs from the oil and surfactant. An increase in particle size of ~300 nm and ~490 nm was recorded for acetone and isopropanol washes, respectively (Table 2 E2, E3). While acetone proved more reliable for the isolation of AlgNPs, this reagent alone was not sufficient in separating AOT from the aqueous phase or maintaining homogeneity of the sample. To improve on this, a 0.2 μm membrane filter and ethyl acetate were used in the wash steps. Passing the sample through this filter after washing with acetone reduced AlgNPs size to 220 nm (E4). The addition of an ethyl acetate wash did not change the size or morphology of AlgNPs (Table 2 E5, Figure 8C), however, it proved to better purify the sample, as AOT displays high solubility in this solvent [39]. Lastly, these two wash steps were combined with an acetone wash, which yielded a small population of AlgNPs with an average size of 142 nm (E6). To increase the monodispersity of this sample, alginate concentrations were adjusted. Increasing the final alginate concentration to 2.5 mg/mL maintained the average size of the nanoparticle but increased the population to 76% of the sample (E7). Raising the final concentration to 5.0 mg/mL resulted in the smallest population exhibiting an average size of 945 nm, with >1 μm particles also present (8).

## 4. Conclusions

Alginate is an excellent candidate for organic NP synthesis. The preparation of short chain alginate as a precursor for NP formulation can be achieved easily with acid hydrolysis or enzymatic breakdown but is more controlled with the latter method. While the ionic gelation method is relatively simple to perform, it exhibits more heterogeneous particle sizes and progressively uncontrolled growth over time, resulting in hydrogels. The concentration landscape of alginate and CaCl_2_ explored suggests that low concentrations of each (0.10 mg/mL alginate; 0.10 mg/mL CaCl_2_) favor the formation of <200 nm AlgNPs. Additionally, more rigorous homogenization methods in the form of a bath sonicator or a homogenizer rotor limit the growth of AlgNPs.

On the other hand, water-in-oil emulsification allows for more controlled size and less polydispersity, as inverse micelles of a defined size restrict the growth of AlgNPs preventing continuous crosslinking and macrostructures. Thus, the optimization of the water:organic phase:surfactant ratio and subsequent purification allowed for a controlled outcome.

As each synthesis method requires different precursor concentrations and presents unique challenges to overcome in purification, it is difficult to compare the viability of the ionic gelation and water-in-oil emulsification method in producing small uniform AlgNPs. However, we have found that careful tuning of parameters allowed for <200 nm AlgNPs to be obtained by either method.

## Figures and Tables

**Table 1:** Experimental parameters and results of the alginate nanoparticle synthesis by ionic gelation. Final alginate and CaCl_2_ concentrations are provided.

Sample ID	Alginate Digest	Alginate (mg/mL)	CaCl_2_ (mg/mL)	Size (nm) % Population	PDI	Homogenization Method/Time
IG1	Acid hydrolyzed	0.095	0.10	227.6 ± 19.7; 100%	0.178	Magnetic stirring (1300 rpm, 45 min)
IG2	Acid hydrolyzed	0.285	0.10	242.2 ± 9.5; 100%	0.155	Magnetic stirring (1300 rpm, 45 min)
IG3	Acid hydrolyzed	0.570	0.10	206.6 ± 3.5; 100%	0.163	Magnetic stirring (1300 rpm, 45 min)
IG4	Enzyme-digested	0.095	0.03	125.9 ± 11.8; 100%	0.617	Magnetic stirring (1300 rpm, 45 min)
IG5	Enzyme-digested	0.095	0.10	118.3 ± 20.1; 100%	0.695	Magnetic stirring (1300 rpm, 45 min)
IG6	Enzyme-digested	0.095	0.55	232.2 ± 5.4; 100%	0.072	Magnetic stirring (1300 rpm, 45 min)
IG7	Enzyme-digested	0.285	0.10	200.9 ± 3.2; 100%	0.17	Magnetic stirring (1300 rpm, 45 min)
IG8	Enzyme-digested	0.285	0.20	571.9 ± 6.7; 100%	0.49	Magnetic stirring (1300 rpm, 45 min)
IG9	Enzyme-digested	0.285	0.40	2826 ± 22.8; 100%	0.44	Magnetic stirring (1300 rpm, 45 min)
IG10	Enzyme-digested	0.095	0.10	108.3 ± 2.5; 100%	0.165	Bath sonication (60 min)
IG11	Enzyme-digested	0.095	0.28	342.5 ± 1.9; 100%	0.247	Bath sonication (60 min)
IG12	Enzyme-digested	1.2	0.10	400.7 ± 4.8; 100%	0.299	Bath sonication (60 min)
IG13	Enzyme-digested	1.2	0.28	319.0 ± 3.1; 100%	0.269	Bath sonication (60 min)
IG14	Enzyme-digested	2.7	0.1	96.56 ± 5.9; 21.1% 722.1 ± 4.7; 41.2%	0.927	Homogenizer (7000 rpm, 35 min)

**Table 2:** Experimental parameters and results of the alginate nanoparticle synthesis by water-in-oil emulsification. Final alginate and CaCl_2_ concentrations in the sample are provided. Emulsions were formed with a water:hexanes:AOT weight ratio of 40:40:20.

Sample ID	(Alginate) (mg/mL); (CaCl_2_) (mg/mL)	Size (nm); % Population	PDI	Washing Method
E1	0.5; 0.6	138.8 ± 13.2; 100%	0.210	N/A
E2	0.5; 0.6	455.3 ± 41.9; 100%	0.492	Acetone
E3	0.5; 0.6	625.5 ± 53.5; 100%	0.423	Isopropanol
E4	0.5; 0.6	220.6 ± 9.2; 100%	1	Acetone + 0.2 μm filter
E5	0.5; 0.6	426.6 ± 38.7; 33.7%	1	Acetone + ethyl acetate
E6	0.5; 0.6	141.7 ± 51.6; 22%	1	Acetone + ethyl acetate + 0.2 µm filter
E7	2.5; 0.6	144.3 ± 20.6; 76%	1	Acetone + ethyl acetate + 0.2 µm filter
E8	5.0; 0.6	945.8 ± 81.3; 32%	1	Acetone + ethyl acetate + 0.2 µm filter

## Data Availability

The data presented in this study are available in the article.
